# Actin cytoskeleton regulator Arp2/3 complex is required for DLL1 activating Notch1 signaling to maintain the stem cell phenotype of glioma initiating cells

**DOI:** 10.18632/oncotarget.16495

**Published:** 2017-03-23

**Authors:** Chen Zhang, Long Hai, Meng Zhu, Shengping Yu, Tao Li, Yu Lin, Bo Liu, Xingchen Zhou, Lei Chen, Pengfei Zhao, Hua Zhou, Yubao Huang, Kai Zhang, Bingcheng Ren, Xuejun Yang

**Affiliations:** ^1^ Department of Neurosurgery, Tianjin Medical University General Hospital, Tianjin, 300052, China; ^2^ Tianjin Neurological Institute, Tianjin, 300052, China; ^3^ Department of Neurosurgery, The Affiliated Hospital of Qingdao University, Qingdao, Shandong, 266003, China

**Keywords:** glioma initiating cell, Notch signaling, delta-like1, cytoskeleton, Arp2/3 complex

## Abstract

Glioblastoma (GBM) is the most common and lethal primary intracranial tumor. Actin cytoskeleton regulator Arp2/3 complex stimulates glioma cell motility and migration, and thus triggers tumor invasion. However, little is known regarding the role of actin cytoskeleton in maintaining the stem cell phenotype. Here, we showed that Arp2/3 complex improved stem cell phenotype maintenance through sustaining the activated Notch signaling. ShRNA targeting Notch ligand Delta-like 1 (DLL1) decreased CD133 and Nestin expression, and impaired the self-renewal ability of CD133+ U87-MG and U251-MG glioma cells, indicating DLL1/Notch1 signaling promoted stem cell phenotype maintenance. Interestingly, inhibiting Arp2/3 complex also induced the similar effect of shDLL1. Silencing DLL1 in the Arp2/3 inhibited CD133+ cells did not further abrogate the stem cell phenotype, suggesting DLL1 function requires Arp2/3 complex in glioma initiating cells (GICs). However, exogenous soluble DLL1 (sDLL1) instead of endogenous DLL1 rescued the Arp2/3 inhibition-induced stem cell phenotype suppression. The underlying mechanism was that Arp2/3 inhibition impeded DLL1 vesicular transport from cytoplasm to cell membrane, which resulted in DLL1 unable to activate Notch pathway. Furthermore, we illustrated that Arp2/3 inhibition abolished the tumorigenicity of CD133+ U87-MG neurosphere cells in the intracranial model. These findings suggested that cytoskeleton maintained the stem cell phenotype in GBM, which provide novel therapeutic strategy that anti-invasive targeted therapies may help eliminate GICs.

## INTRODUCTION

Glioblastoma (GBM; World Health Organization grade IV) is the most common and lethal primary intracranial tumor [1]. GBM exhibits a relentless malignant progression characterized by widespread invasion throughout the brain, resistance to chemical and radiation therapeutic approaches, and tumor recurrence. Without treatment, most patients will die of their disease within 3 months of diagnosis [1, 2]. Surgical intervention can extend survival to 9 to 10 months, and this can be lengthened to 12 months with the addition of adjuvant radiation [1, 2]. In spite of advances in targeted therapies and immunotherapies, the recent standard-of-care treatment consisting of maximal surgical resection, followed by radiotherapy with concomitant and adjuvant temozolomide (TMZ), just prolongs the median survival period of GBM patients to 14.6 months [2]. The incorporation of bevacizumab, a recombinant humanized monoclonal antibody that blocks angiogenesis by inhibiting vascular endothelial growth factor A (VEGF-A), to the standard-of-care treatment led to significant improvements in progression-free survival rates but not in overall survival duration [3]. Even worse, bevacizumab may lead to an apparent phenotypic shift to a predominantly infiltrative pattern of tumor progression [4]. The other phase III clinical trial completed targeted therapy-cilengitide also did not improve outcomes [5]. Therefore, novel therapeutic strategies are imperative for the treatment of GBM patients.

Several groups in parallel demonstrated that gliomas contain self-renewing and multipotent GICs, which are resistant to radiation and chemotherapy, and can lead to tumor recurrence [6, 7]. GICs possess the capacity to generate differentiated glioma cells through asymmetric cell division and to form intracranial xenograft *in vivo* [8]. In this study, we applied CD133 and Nestin to label GICs. Notch signaling pathway plays a critical role in promoting stem cell fate and affecting GICs maintenance [9]. Notch signaling is an evolutionarily conserved pathway, which participates in cell fate decision, differentiation, survival, angiogenesis, and migration [10–12]. In mammals, Notch pathway consists of five trans-membrane ligands (Delta-like 1, 3 and 4 and Jagged 1 and 2) and four membrane bound receptors (Notch 1, 2, 3 and 4). As one of the most profoundly studied Notch ligands, Delta-like1 (DLL1) has been reported to enhance cancer cell stemness, tumorigenicity, metastasis, and keep cancer stem cells in the undifferentiated status [13–16]. In spite of diverse activating mechanisms, the canonical Notch signaling begins upon Notch ligand binding to the extracellular domain of Notch receptor through local cell-cell interactions [17]. When receptors are triggered by ligands, it promotes two proteolytic cleavage events at receptors. The cleaved Notch intracellular domain (NICD, activated form of Notch) relocates to the nucleus, where it interacts with the DNA-binding protein RBPJk, activating a transcriptional complex known as CSL and then resulting in transcription of targeting genes, such as Hes1, Hes3, Hes5, Hey1, and Hey2.

Actin-related protein2/3 complex (Arp2/3 complex, ArpC) is one major regulator of the actin cytoskeleton [18]. It is composed of seven subunits that act together to nucleate new actin filaments off of pre-existing actin filaments [19]. In cultured motile cells, where roles for ArpC are intensively studied, ArpC stimulates the formation of new branched actin filaments, producing pseudopodia, further pushing the membrane forward for cell migration [19, 20]. In glioma, ArpC is elementary for tumor cell motility and tumor invasion [21]. Rajan et al. have illustrated that ArpC is required for Notch ligand Delta trafficking in *Drosophila* development [22], as actin cytoskeleton serves as “highways” for intracellular vesicular transport. In this study, we assume that ArpC regulates Notch component transport, and thus engages in stem cell phenotype maintenance.

Here, we showed that Delta-like1 (DLL1) activated Notch1 signaling to maintain the stem cell phenotype of GICs. Silencing DLL1 decreased expression of stem cell markers and impaired self-renewal ability in GICs. ArpC was required for DLL1 vesicular transport from cytoplasm to cell membrane, and thus was involved in regulating Notch1 activity and maintaining stem cell phenotype.

## RESULTS

### CD133+ glioma neurospheres exhibited high DLL1 expression and notch activity

To study the mechanism underlying stem cell phenotype maintenance of GICs, we established CD133+ glioma neurosphere model *in vitro*. Then, we applied magnetics activated cell sorting (MACS) to enrich CD133+ cells from U87-MG and U251-MG glioma cells. To confirm the effectiveness of MACS, flow cytometry was performed to test the percentage of CD133+ cells in MACS+ population. Before sorting, there was only 2.05±1.36% CD133+ cells in U87-MG and 2.36±1.20% in U251-MG cells (Supplementary Figure 1A). After sorting, the percentage of CD133+ cells (84.70±2.70% in U87-MG and 74.23±2.43% in U251-MG) was significantly increased in MACS+ proportion (Supplementary Figure 1A). Then, the CD133+ cells were cultured in stem cell medium to form neurospheres, while the cells without CD133 sorting were unable to develop spheroids in the culture (Supplementary Figure 1B).

**Figure 1:**
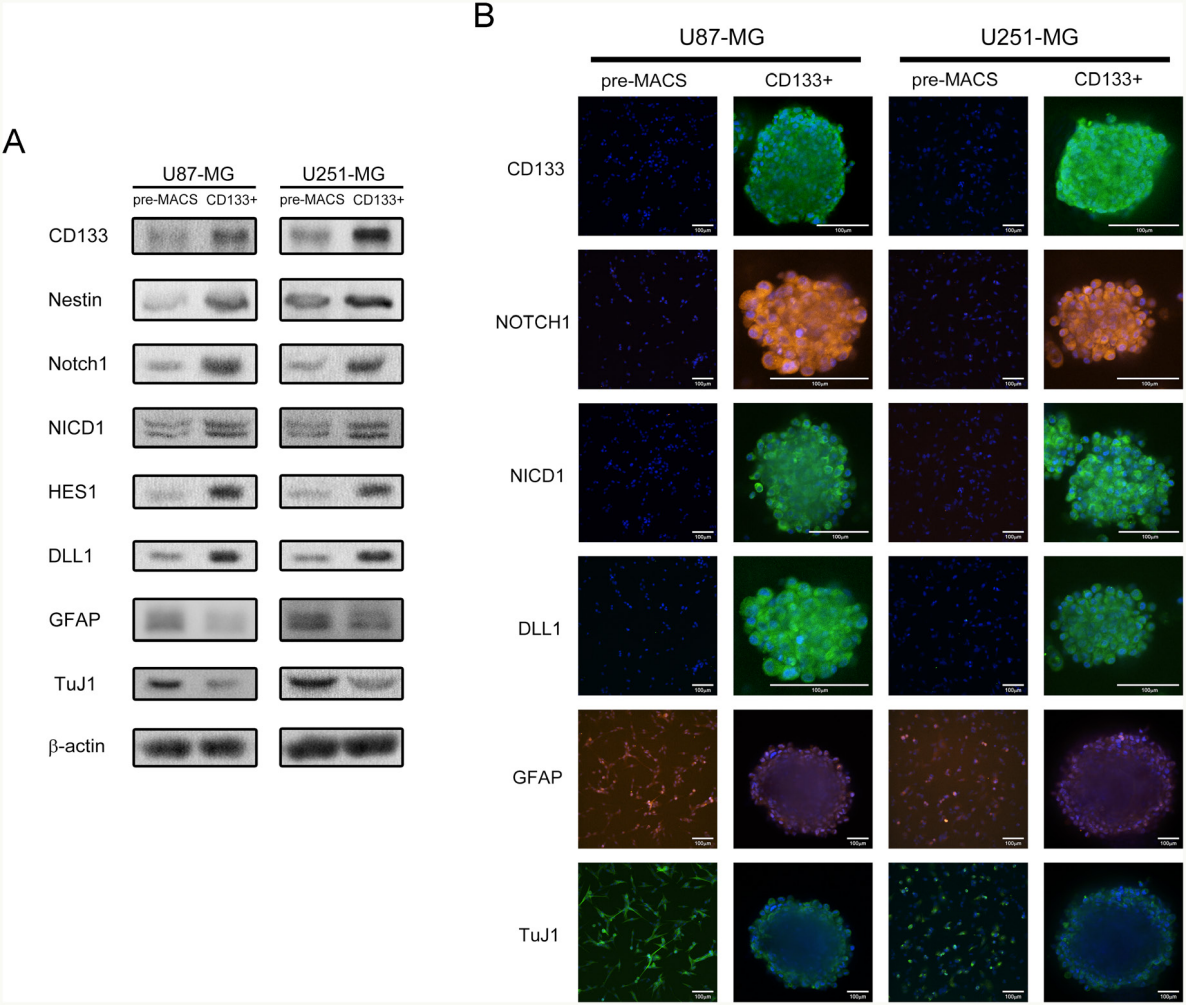
CD133+ U87-MG and U251-MG human GBM formed neurospheres exhibit higher stem cell marker expression, Notch activity, and elevated self-renewal abilities **(A)** The protein expression of pre-MACS and sorted CD133+ cells. **(B)** Immunofluorescence staining of pre-MACS cells and sorted CD133+ neurospheres. Images were captured by laser confocal microscope.

Both CD133 and Nestin were selected to assess the stem cell phenotype. Notch1 signaling activity was evaluated through NICD1 and target gene HES1. Cell differentiation was detected by glial fibrillary acidic protein (GFAP, astrocyte marker) and class-III beta-tubulin (TuJ1, neuronal marker). Western blot and immunofluorescence staining displayed that CD133+ neurospheres highly expressed stem cell markers (CD133 and Nestin), DLL1, Notch1, and activated Notch components (NICD1 and HES1). Differentiation markers GFAP and TuJ1 were less expressed in CD133+ neurospheres (Figure 1A and 1B). These results revealed that CD133+ glioma neurosphere model *in vitro* enriched self-renewal GICs with highly activated Notch signaling.

### DLL1 maintained the stem cell phenotype of GICs

**Figure 2:**
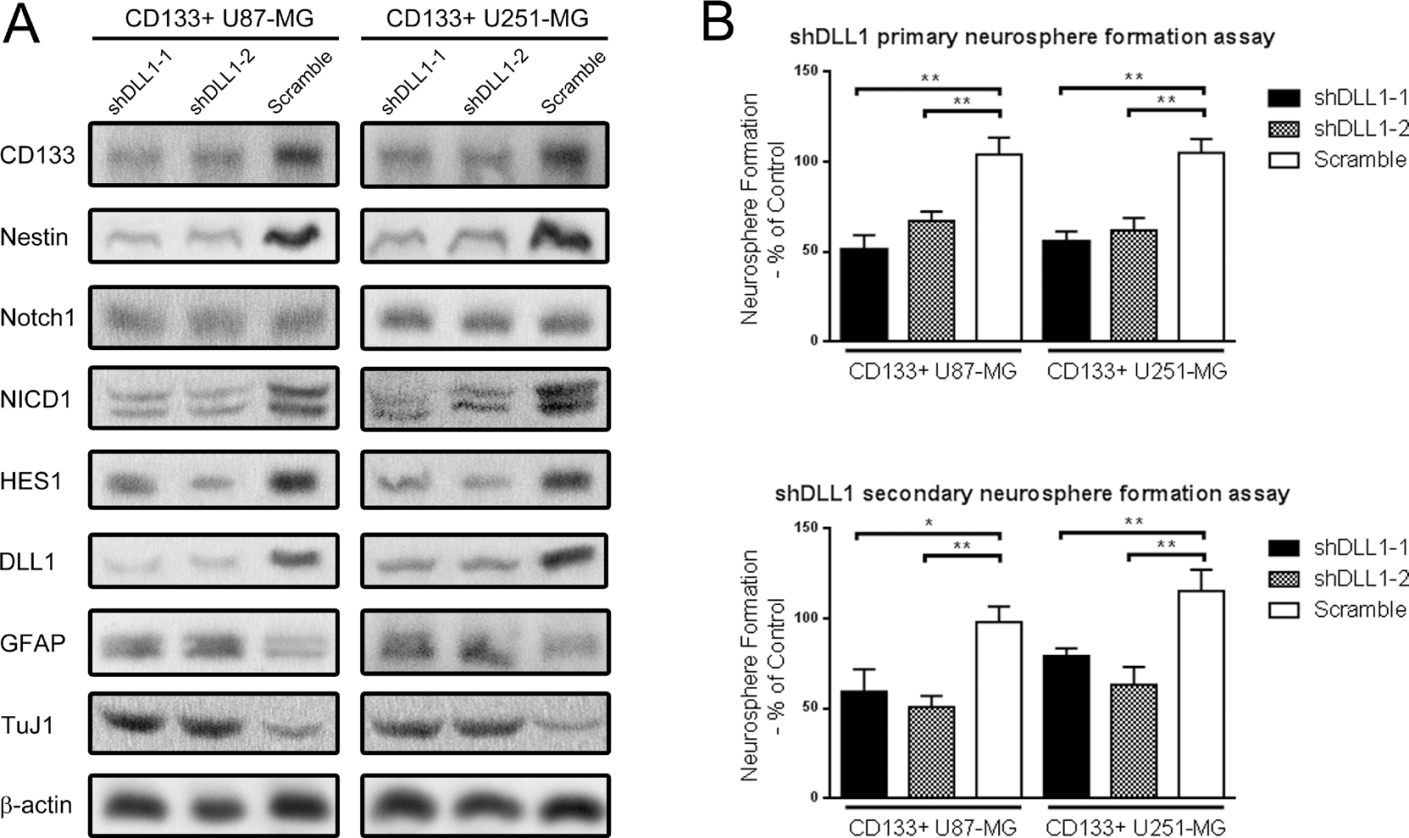
DLL1 silencing decreased stem cell markers expression and Notch activity, and impaired the self-renewal ability of CD133+ U87-MG and U251-MG glioma neurospheres **(A)** Two shRNAs targeting Notch ligand DLL1 and one scramble shRNA were transfected into CD133+ neuropshere cells. Protein expression was detected through Western blot. **(B)** Primary and secondary single cell neurosphere formation assay against shDLL1 CD133+ cells. (*: p<0.05, **: p<0.01).

Notch ligands and receptors are both trans-membrane proteins. The canonical activating way of Notch in signal-receiving cells requires Notch ligands expressed signal-sending cells, in which Notch ligand on sending cells activates Notch on receiving cells through cell contact. To clarify whether DLL1 contributed to maintaining the stem cell phenotype, shRNAs targeting DLL1 were transfected into CD133+ U87-MG and U251-MG glioma neurosphere cells. We found that shDLL1 decreased stem cell markers CD133 and Nestin expressions in neurospheres, while cell differentiation markers GFAP and TuJ1 were more highly expressed. NICD1 and HES1 expression were also downregulated in shDLL1 neurospheres. However, Notch1 expression was not affected by shDLL1, implying that DLL1 regulated Notch1 signaling activity instead of Notch1 expression (Figure 2A). Meanwhile, shDLL1 significantly diminished primary and secondary neurosphere formation frequencies than scramble cells (p<0.05) (Figure 2B). Above all, silencing DLL1 impaired self-renewal ability and decreased stem cell marker expressions demonstrated that DLL1 was involved in maintaining the stem cell phenotype.

### ArpC inhibition impaired the stem cell phenotype

**Figure 3:**
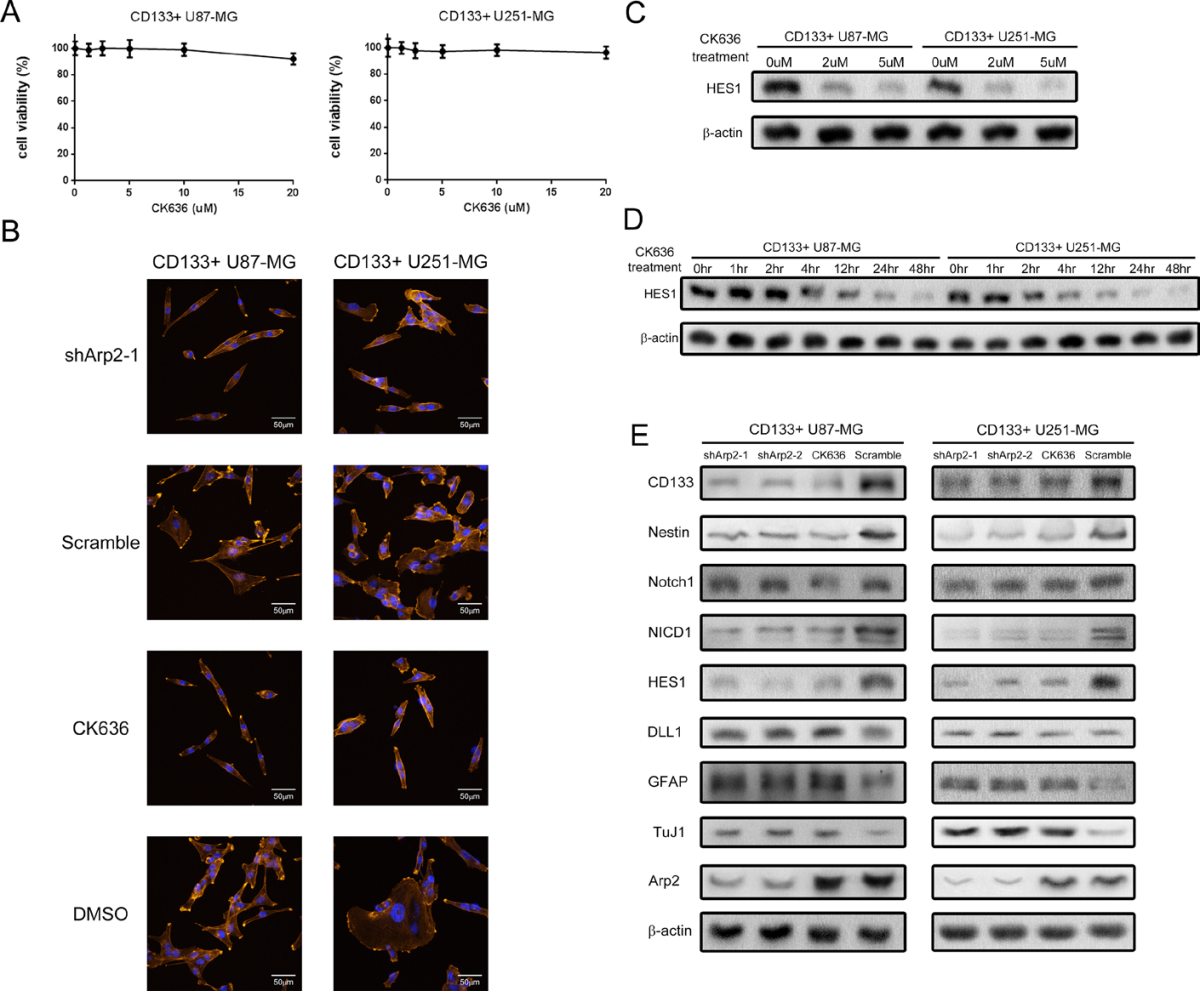
ArpC inhibition impaired the stemness marker expression, Notch activity, and self-renewal ability of CD133+ U87-MG and U251-MG glioma neurospheres **(A)** Cells were treated by ArpC specific inhibitor CK636 for 24 hours. Cell viability was determined by Trypan Blue assay. **(B)** CD133+ cells were cultured in 10% serum-containing medium to induce lamellipodia. The suppression of lamellipodia formation was observed through confocal microscope after CK636 (2μM) treatment for 30 minutes (Orange: actin filaments, Blue: Nucleus). **(C)** HES1 expression was determined after CK636 treatment for 24 hours with different doses. **(D)** HES1 expression was measured after CK-636 (2μM) treatment for different periods. **(E)** Protein expression was detected by Western blot after shArp2 and CK636 (2μM) treatment for 24 hours.

ArpC is crucial for regulating the cytoskeleton and the formation of lamellipodia, which stimulates glioma cell invasion and migration [21]. To study whether ArpC engages in maintaining GICs stem cell phenotype, we inhibited the function of ArpC through using both subunit Arp2 shRNAs and specific inhibitor CK636, which stabilizes the inactive conformation of subunits [23]. To define the minimum effective dose of CK636, we cultured CD133+ cells in 10% serum-containing medium to induce attached cells so that to inspect lamellipodia formation, which indicates ArpC function. We applied serum medium here due to that the morphological change of cytoskeleton is hardly observed in non-attached round cells cultured with stem cell medium. We found that CK636 (2uM) treatment significantly restrained lamellipodia formation without decreasing cell viability, illustrating ArpC function was suppressed (Figure 3A and 3B). In stem cell medium culture, we employed 2uM dose of CK636 treatment for 24 hours, which decreased HES1 expression significantly, to further investigate the influence of ArpC on stem cell phenotype (Figure 3C and 3D). Interestingly, both shArp2 and CK636 treatments induced comparable protein expression and neurosphere formation alternations with silencing DLL1. Arp2 knockdown also impaired the self-renewal ability of CD133+ neurospheres, implying that ArpC was involved in maintaining stem cell phenotype (Figure 3E).

### Exogenous DLL1 rescued ArpC inhibition-induced stem cell phenotype abrogation

**Figure 4:**
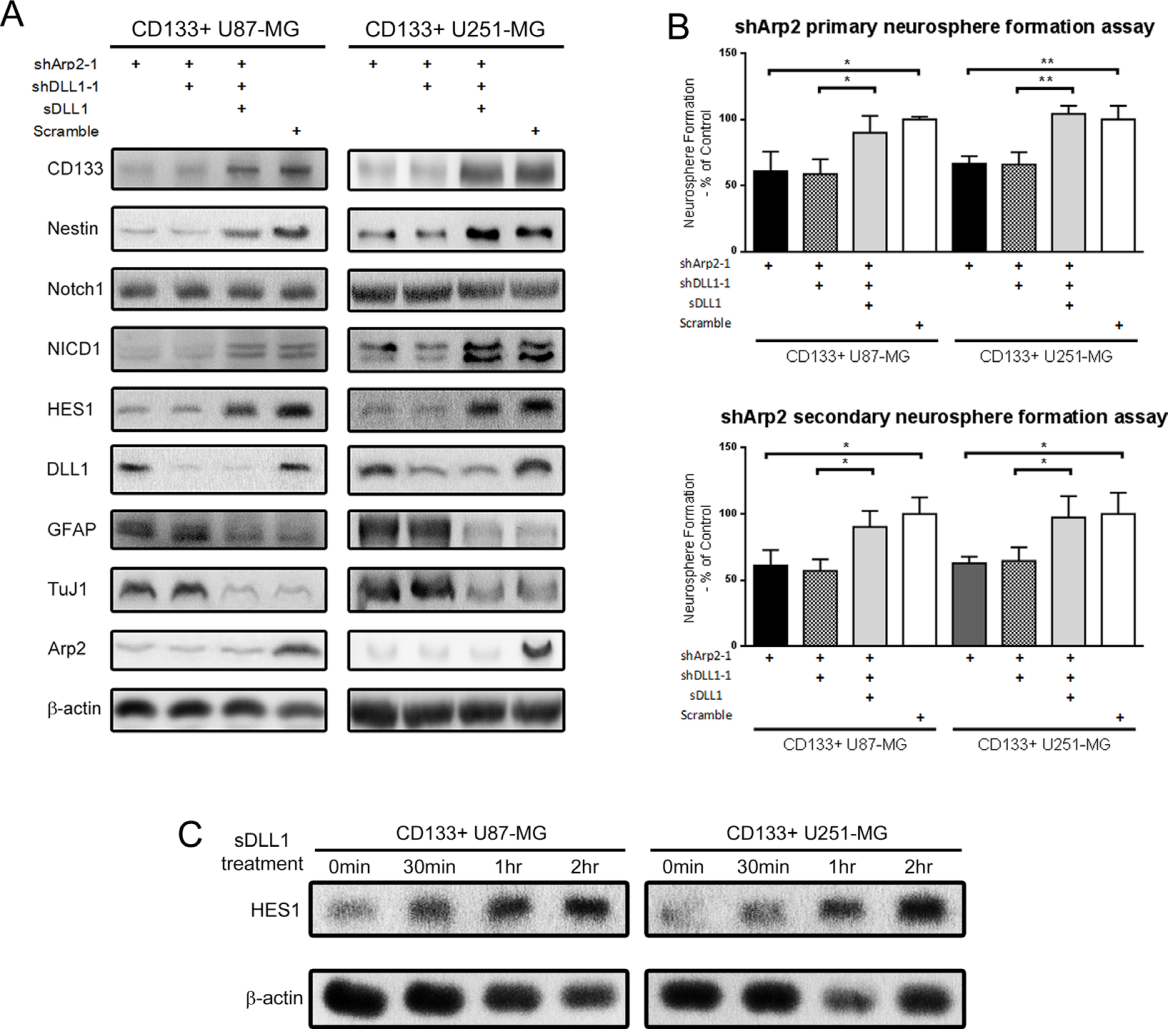
Soluble DLL1 instead of endogeneous DLL1 rescued the impaired stem cell phenotype induced by Arp2 silencing in CD133+ U87-MG and U251-MG cells **(A)** Protein expression after shArp2, shDLL1, and soluble DLL1 treatment. **(B)** Primary and secondary single-cell neurosphere formation assay after shArp2, shDLL1, and soluble DLL1 treatment. (*: p<0.05, **: p<0.01) **(C)** Cells were treated with 5μg/ml soluble DLL1for different periods. HES1 expression was detected.

According to the above results, we silenced both Arp2 and DLL1 and suppose to observe synergistic effect on stem cell phenotype. However, CD133+ cells did not exhibit any synergistic or additive effect with silencing both Arp2 and DLL1 (Figure 4A and 4B). Silencing DLL1 did not further abrogate stem cell markers expression and self-renewal capacity in shArp2 CD133+ neurospheres, revealing that ArpC inhibition abolished DLL1 function. DLL1 required ArpC to maintain the stem cell phenotype. Interestingly, exogenous soluble DLL1 instead of endogenous DLL1 was able to rescue the shArp2-induced stem cell phenotype abrogation (Figure 4A and 4B). Soluble DLL1 treatment for 2 hours, which induced HES1 expression significantly, was applied in this experiment (Figure 4C). The above data made us pose the hypothesis that ArpC regulated DLL1 subcellular localization. ArpC might participate in protein vesicular transport from cytoplasm to cell membrane.

### ArpC regulated the transport of DLL1 vesicles from cytoplasm to cell membrane

**Figure 5:**
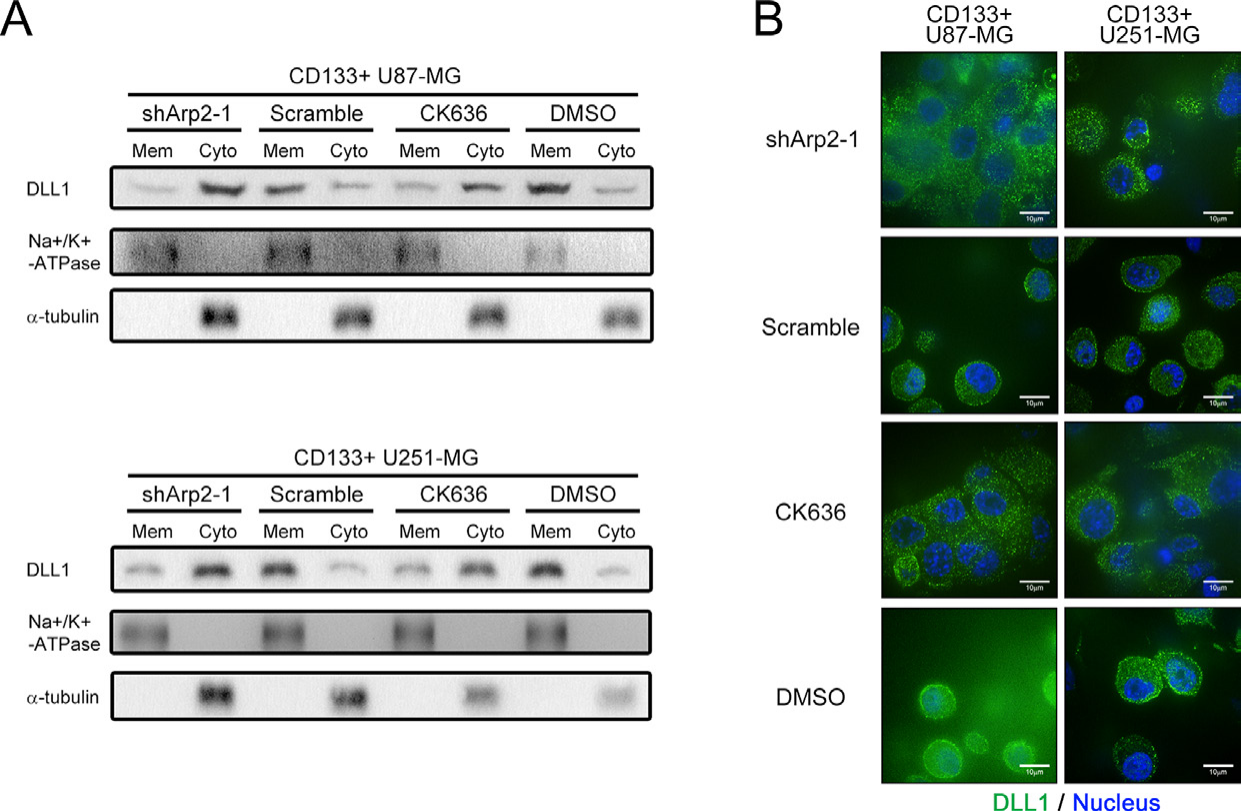
ArpC maintained the subcellular localization of DLL1 on membrane in CD133+ U87-MG neurosphere cells **(A)** Membrane and cytoplasmic proteins were extracted separately. Sodium potassium ATPase and a-tubulin served as membrane (Mem) and cytoplasmic (Cyto) loading control, respectively. DLL1 expression was detected. **(B)** DLL1 expression of shArp2 and CK636-treated cells was immunofluorescence stained for confocal microscope observation. Green: DLL1. Blue: Nucleus.

To further verify our hypothesis that DLL1 localization was affected, we harvested cytoplasmic and membrane-enriched protein fractions by Subcellular Protein Fraction Kit for western blot. Indeed, there was more DLL1 presented in cytoplasmic fraction but less in cell membrane-enriched fraction after ArpC inhibition (Figure 5A and 5B). Consistent with the Western blot data, the confocal immunofluorescence result also manifested more DLL1 localized in cytoplasm in ArpC inhibited CD133+ cells (Figure 5C). The finding further confirmed that ArpC was involved in DLL1 vesicle transport, which resulted in more DLL1 accumulated in cytoplasm but less presented on membrane, and thus unable to activate Notch signaling to maintain stem cell phenotype.

### ArpC inhibition abolished GICs tumorigenicity

**Figure 6:**
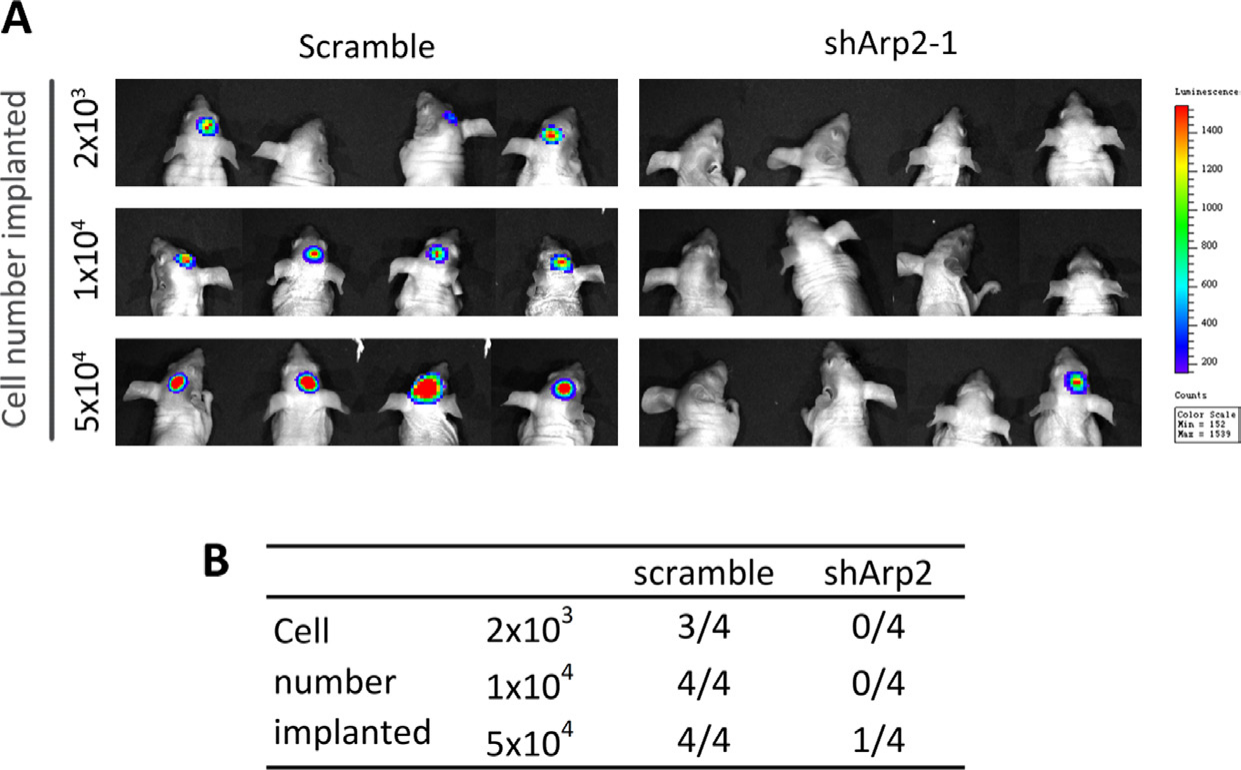
Silencing Arp2 impaired the tumorigenicity of CD133+ U87-MG neurosphere cells *in vivo* Different amount of cells (2×10^3^, 1×10^4^, 5×10^4^) were implanted into the brain of nude mice. **(A)** Image of the formed xenografts at 42 days after implantation. **(B)** ShArp2 decreased the incidences of tumor formation in intracranial xenograft models.

*In vivo*, tumorigenicity is commonly utilized to evaluate stem cell phenotype. To study the function of ArpC in tumorigenicity, we performed tumor formation titration assay *in vivo*. Tumorigenicity was determined through both xenograft formation incidences. ShArp2 and scramble CD133+ U87-MG neurosphere cells were implanted into the brain of the nude mice (Supplementary Figure 2). Arp2 knockdown cells exhibited fewer incidences to form xenografts than scramble cells at 42days after implantation (Figure 6A and 6B). These results elucidated that ArpC was critical for the stem cell phenotype maintenance *in vivo*.

## DISCUSSION

Notch signaling has been implicated in a growing number of hematopoietic and solid tumors. Inappropriate Notch activation restricts differentiation and/or prevents apoptosis [24]. In gliomas, Notch signaling has been shown to maintain the stem cell phenotype. Notch inhibition induces CD133+ population ablation, increased differentiation, reduced clonogenic potential, and impaired tumorigenicity [25, 26]. In this study, we pointed out that DLL1 was required for Notch activity to maintain glioma stem cell phenotype. Importantly, actin cytoskeleton engaged in the ligand-dependent Notch activation. Cytoskeleton regulator ArpC participated in the vesicular transport of DLL1 from cytoplasm to cell membrane, and thus ensuring DLL1 to activate Notch signaling.

For the stem cell markers, a number of markers have been proved useful for the isolation of tumor-initiating cells in GBM [27]. Notably, tumor-initiating cells have frequently been enriched using markers specific for normal stem cells of the same organ. None of these markers are exclusively expressed by tumor-initiating cells, highlighting the necessity to use combinatorial markers [28]. Singh et al. demonstrated that transplantation of as few as 100 human GBM-derived CD133+ cells into mouse brains gave rise to a tumor [8]. Therefore, CD133 appears to be a robust cell surface marker for GICs. Since then, numerous studies applied CD133 to detect GICs. Similarly, the increase of Nestin expression appeared to be a common phenomenon associated with the stem cell phenotype of cells within the sphere. Studies reported the co-expression of Nestin with CD133 as a common GIC phenotype in tumors of neurogenic origin and utilized Nestin and CD133 to delineate GICs [8, 29–32]. In this paper, CD133 and Nestin were introduced to label GICs combinatorically, even though they are expressed in both GICs and neural stem cells [8, 30, 33].

GICs reside in the stem cell niches to sustain their self-renewal and undifferentiated state [34]. Niches are specialized microenvironments that regulate stem cell fate by providing cues in the form of both cell-cell contacts and secreted factors. Zhu et al. showed that endothelial cells expressing Notch ligand created a stem cell niche to maintain the stem cell phenotype [35]. Meanwhile, several publications illustrated that GICs also contributed to the vasculogenic mimicry [36–39]. CD133+ GBM cells could differentiate into endothelial-like cells to provide blood supply [36–39]. However, these vasculogenic mimicry were met with some criticisms. There were serious technical limitations in identifying endothelial cells versus tumor cells in contact with the vascular lumen [40, 41]. It was also unclear whether there was a connection between endothelial cells and tumor cells in blood vessel walls [40, 41]. Further investigation is required to explore whether the Notch pathway ligands and receptors expression pattern is different in stem cell niches and in vasculogenic mimicry [35].

An original method was introduced in our study to stain cells inside neurosphere. To our knowledge, there are not many publications of protein expression pattern of cells inside a spheroid. Immunofluorescence staining always fails to label an entire spheroid, due to the suspended characteristic of the sphere. Compared with other existed spheroid staining methods [32, 42–49], our approach maximally preserves the intact composition and morphology of spheres, and thus provides the most accurate readout of the entire sphere. Even the expression pattern of cells with low cell viability can also be observed, and no cell is removed by centrifuging or low adherence. Importantly, it realizes the detection of cell subgroups and cell-cell interactions inside a sphere, which could help further our understanding and research regarding spheres and stem cells.

In this study, we showed that DLL1 was important on maintaining glioma stem cell phenotype. But DLL1 required Notch signaling to perform its function. The variable expression of molecules downstream of DLL1, such as Notch1, Notch2, HES1, and HES5, among patients could affect the function of DLL1 on stem cell phenotype [50]. Additionally, the subcellular localization of DLL1 was also critical to its function. Thus, DLL1 expression could not be used to evaluate patient survival, although the stem cell phenotype was correlated with clinical outcome in GBM [51].

Our finding elucidated that Notch activity is more dependent on the canonical ligand binding in glioma, although the non-canonical activating mechanisms have been reported, including the bypass through Wnt pathway or in a ligand-independent manner [17, 52]. Other mechanisms may perform their function in glioma, but they are not sufficient to determine Notch activity. Among the five ligands, it has been shown that silencing Notch ligands abrogated the clonogenic potential of GBM cells *in vitro* and tumorigenicity *in vivo* [35, 53]. Here, we illustrated the critical role of DLL1 in the stem cell phenotype maintenance. DLL1 may serve as a potential target to eliminate GICs.

Cytoskeleton functions as “highways” for the intracellular traffic. Before performing their functions, nascent proteins are processed by the endoplasmic reticulum (ER) and the Golgi complex, and then are stored in specialized vesicles which fuse with cell surface [54–57]. Actin cytoskeleton plays an essential role in the vesicular transport [57, 58]. In Drosophila melanogaster, ArpC is required for Notch ligand Delta trafficking during development [22]. Here, we clarified that ArpC participated in Notch signaling through promoting the transport of ligand DLL1 to membrane (Figure 6). To our knowledge, the role of cytoskeleton in Notch signaling in human cancers has not been described previously. Notably, our study introduced ArpC as one of the major cytoskeleton mediators to verify our hypothesis that cytoskeleton participated in stem cell phenotype maintenance. It requires further investigation to figure out whether other targets participate in actin filaments formation [59], and then mediating stem cell phenotype.

Numerous studies reported that CSCs were resistant to radiation and chemotherapy, and resulted in tumor recurrence [60]. We demonstrated that silencing Notch ligand eliminated GICs, which required cytoskeleton regulator ArpC. Our previous data have shown that ArpC was responsible for glioma cell invasion and migration [21]. As anti-invasive therapies are under intense investigation recently [61], we suggested that anti-invasive therapy also help eliminate the stem cell phenotype in gliomas. Future work would include validations in multiple independent studies, the development of reliable molecular assays for clinical samples, and discoveries of selective inhibitors with less toxicity and better brain penetration. Overall, the better understanding of the cellular and molecular regulatory mechanisms between CSCs and tumor invasion may lead to rational new therapies for cancers.

## MATERIALS AND METHODS

### Immunofluorescence analysis

An original method was applied to label proteins in the glioma neurospheres. Neurospheres were placed into cell insert (Millipore, US) and fixed by 0.4% Paraformaldehyde solution (Solarbio, China). Then, the neurospheres were washed three times in PBS, and incubated with primary antibodies (See Supplementary Table 1) overnight at 4°C. Alexa-Fluor 488 conjugate anti-rabbit secondary antibody (1:5000, Cell signaling, US) and Alexa-Fluor 594 conjugate anti-mouse secondary antibody (1:5000, Life technologies, US) were used for fluorescent double-staining. Serum medium cultured CD133+ cells were stained through Alexa-Fluor 594 conjugate phalloidin (1:200, Life technologies, US) for 20 minutes at 37°C to exhibit actin cytoskeleton. DAPI solution (Solarbio, China) was employed to label cell nucleus. Images were observed and captured by Perkinelmer UltraVIEW VOX confocal microscope (Institute of Hematology and Blood Diseases Hospital, Chinese Academy of Medical Sciences and Peking Union Medical College, Tianjin, China).

### Cell lines

U87-MG and U251-MG glioma cells were obtained from the American Type Culture Collection (ATCC). Cells were cultured in DMEM medium containing 10% FBS (Gibco, US). After MACS, CD133+ cells were cultured in stem cell medium (DMEM/F12 medium supplemented with 10ng/ml EGF, 10ng/ml bFGF, and B27 (1:50, Invitrogen, US)). The neurosphere can be observed at the second day.

### Magnetic activated cell sorting and flowcytometry analysis

CD133+ glioma cells were collected by CD133 MicroBead Kit (Miltenyi, Germany) following manufacturer's protocol. The collected cells were stained by anti-human CD133 antibody (Miltenyi, Germany) at 4°C overnight and then Alexa Fluor 488 conjugate anti-mouse secondary antibody. The percentage of CD133+ cells was analyzed by BD FACS Caliber, Aria III (Tianjin Neurological Institute, Tianjin, China).

### Western blot

Cells were lysed in the RIPA buffer (Solarbio, China) with PMSF (1:100, Solarbio, China). And the total protein concentration was determined using the BCA Protein Assay Kit (Solarbio, China), according to the manufacturer's instructions. Samples were analyzed by gel electrophoresis, blotted to PVDF membrane (Millipore, US), and probed using primary antibodies (See Supplementary Table 1) followed by the HRP-conjugated goat anti-mouse or rabbit IgG antibodies (ZSGB-Bio, China). β-actin (1:5000, Solarbio, China) was utilized as loading control. The membrane was developed using the Luminata Classico Western HRP substrate (Millipore, US). Membrane and cytoplasmic proteins were extracted by Subcellular Protein Fraction Kits (Thermo Scientific, US). Sodium potassium ATPase and α-tubulin (See Supplementary Table 1) were utilized as loading control for membrane and cytoplasmic proteins, respectively.

### Single-cell neurosphere formation assay

For primary single-cell neurosphere formation assay, after 24 hours treatment with the lentivirus, cells were disassociated and single-cell suspension was cultured in 96-well plates (one cell per well) containing 100ul supplemented stem cell medium. After 21 days, the percentage of wells with neurosphere was quantified. For secondary neurosphere formation assay, primary neurospheres were dissociated into single cells and were seeded in 96-well plates again. The percent of wells with secondary neurospheres was counted after 21 days.

### Cell viability assay

The cell viability was estimated using 0.4% trypan blue exclusion test (Sigma-Aldrich, US), according to manufacturer's guidelines. Cells were treated with different doses of CK636 for 24 hours. 900μl cell suspension was mixed with 100μl 0.4% trypan blue solution. Following incubation for 3 minutes at room temperature, cells were loaded on a slide for microscope observation. The percentage of viable cells was calculated by viable cell numbers / (viable cell + dead cell numbers) × 100%. At least 400 cells were counted to assess cell viability for each sample.

### Lentiviral transfection

Lentiviral shRNA constructs were obtained from Genechem Co., Ltd., China. ShRNA sequences were as follows: GGACCTGAACTACTGCACA (shDLL1-1), CCTTCTCTCTGATTATTGA (shDLL1-2), GGAGGGATATAACTAGATA (shArp2-1), and GCATAGTACGAAATTGGG (shArp2-2). Firefly Luciferase lentiviral particles were obtained from Genecopoeia, China. Cells were transfected with either lentiviral particles, following the manufacturer's recommendations. After infection, stable cell clones expressing the shRNA constructs were isolated by selection with 5μg/ml puromycin solution. Cells were collected for further experiments at 48 hours after the transfection. For double knockdown, another shArp2-1 with neomycin resistance (QIAGEN, US) was transfected in shDLL1-1cells. And DLL1 and ARP2 silencing cells were selected by 500μg/ml G-418.

### Soluble DLL1 and CK636 treatment

For soluble DLL1 treatment, cells were collected after 2 hours treatment with 5μg/ml recombinant human DLL1 (Enzo Life Sciences, US). Cells were fixed after 30 minutes CK636 (Selleckchem #S7497, US) treatment for cytoskeleton structure assessment, and total protein was extracted after 24 hours treatment for expression detection.

### Animals and intracranial xenograft model

Animal experiments were approved by the Ethical Committee in Tianjin Medical University General Hospital. Totally 24 female immunocompromised nude mice, aged 4 weeks, were randomly divided into 2 groups (12 mice each group) for intracranial implantation of shArp2 and scramble CD133+ U87-MG neurosphere cells with luciferase expression, respectively. Each group was randomly divided into 3 subgroups (4 mice each subgroup) inoculated with different number (2×10^3^, 1×10^4^, and 5×10^4^) of cells. Mice were anaesthetized, placed in a stereotactic frame (RWD life science, China), and injected with specific numbers of glioma neurosphere cells in 10μl of PBS through a 27-gauge needle at 2mm lateral and posterior to the bregma and 3mm below the dura. Cell suspension was injected slowly in 20 minutes. Then the needle was kept in the injection site for 5 minutes before removing it. Mice were housed under pathogen-free conditions in the barrier animal facility. Tumor cells bioluminescence imaging was performed to assess xenograft formation at 2 days and 42 days after implantation by using the IVIS Spectrum Live Imaging System (Tianjin Medical University, China). Image calibration and visualization were performed using Live Image 4.4 Software.

### Statistical analysis

All quantified data represent an average of at least triplicate experiments unless otherwise indicated, and standard deviations were calculated. All statistical analyses were performed using GraphPad Prism 6.0 (GraphPad Software, La Jolla, CA, USA). Comparisons among groups were performed using unpaired Student's t-tests. P<0.05 was considered to be statistically significant.

## SUPPLEMENTARY MATERIALS FIGURES AND TABLES


