# Supplementary material for: Actin cytoskeleton regulator Arp2/3 complex is required for DLL1 activating Notch1 signaling to maintain the stem cell phenotype of glioma initiating cells
Source: Oncotarget. 2017 Mar 23;8(20):33353–64. doi: 10.18632/oncotarget.16495 (PMC5464873; doi:10.18632/oncotarget.16495)
Supplement: Supplementary file 1 [file oncotarget-08-33353-s001.pdf]

# Actin cytoskeleton regulator Arp2/3 complex is required for DLL1 activating Notch1 signaling to maintain the stem cell phenotype of glioma initiating cells

## SUPPLEMENTARY MATERIALS

## SUPPLEMENTARY FIGURES AND TABLE

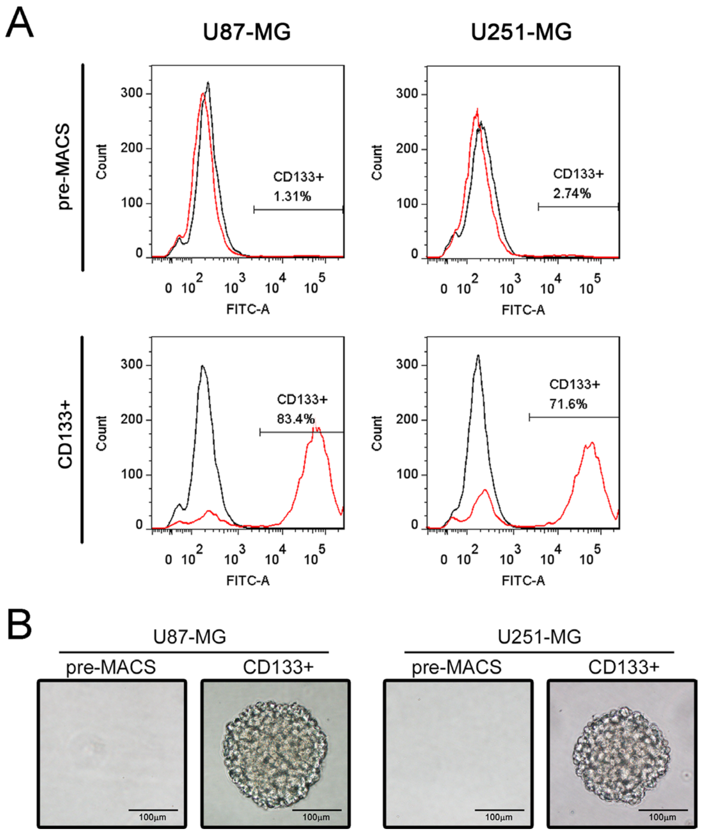


**Supplementary Figure 1: MACS enriched CD133+ glioma neurospheres. (A)** The percentage of CD133+ GBM cells was tested through flow cytometry. Black: negative control. Red: treated cells. **(B)** Pre-MACS and CD133+ cells were cultured in stem cell medium. Images displayed the CD133+ cells formed neurosphere at day 10.

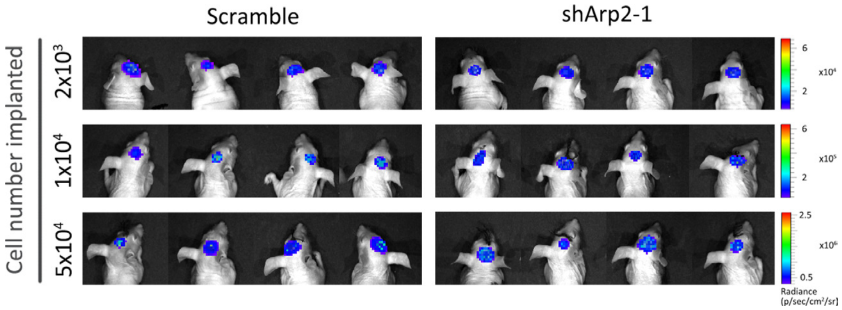


**Supplementary Figure 2: ShArp2 and scramble CD133+ U87-MG neurosphere cells were implanted into the brain of nude mice.** Images were captured at 2 days after implantation.

**Supplementary Table 1: Information of primary antibodies used in this study**

| Target | Manufacturer | Source | Species reactivity | Dilution for IF | Dilution for Western blot |
|---|---|---|---|---|---|
| CD133 | Abcam #ab19898 | Rabbit | Human | 1/100 | 1/500 |
| Nestin | Abcam #ab22035 | Mouse | Human | | 1/1000 |
| Notch1 | Abcam #ab52627 | Rabbit | Human | 1/100 | 1/1000 |
| NICD1 | Cell Signaling #4147 | Rabbit | Human | 1/100 | 1/1000 |
| HES1 | Abcam #ab108937 | Rabbit | Human | | 1/10000 |
| DLL1 | Abcam #ab57308 | Mouse | Human | 1/100 | 1/500 |
| GFAP | Abcam #ab33922 | Rabbit | Human | 1/100 | 1/1000 |
| TUJ1 | Abcam #ab14545 | Mouse | Human | 1/100 | 1/2000 |
| Sodium Potassium ATPase | Abcam #ab76020 | Rabbit | Human | | 1/100000 |
| α-tubulin | Abcam #ab7291 | Mouse | Human | | 1/10000 |
| β-actin | ZSGB-Bio #TA-09 | Mouse | Human | | 1/5000 |

IF: Immunofluorescent staining.
